# Contextualizing marital dissatisfaction: examining profiles of discordant spouses across life domains

**DOI:** 10.3389/fpsyg.2025.1458129

**Published:** 2025-03-19

**Authors:** Annie Regan, Lisa C. Walsh, Calen Horton, Anthony Rodriguez, Victor A. Kaufman

**Affiliations:** ^1^Department of Psychology, University of California, Riverside, Riverside, CA, United States; ^2^Department of Psychology, University of California, Los Angeles, Los Angeles, CA, United States; ^3^Arkoda Research Group, Anchorage, AK, United States; ^4^RAND Corporation, Boston, MA, United States

**Keywords:** marital satisfaction, life satisfaction, friendship satisfaction, family satisfaction, latent profile analysis

## Abstract

Research suggests that up to a third of married individuals report low marital satisfaction, underscoring the importance of studying unhappy marriages. Although numerous studies have investigated the causes and consequences of marital dissatisfaction, less is known about the potential heterogeneity among individuals within unhappy marriages and the extent to which some unhappily married spouses may be satisfied in other life domains. The present study sought to determine whether categorical differences exist among unhappily married individuals. Using friendship satisfaction, family satisfaction, and life satisfaction as indicator variables, we conducted a latent profile analysis (LPA) on married individuals (*N* = 1,070). Specifically, we conducted LPA on subsets of participants reporting the lowest 20%, 22.5%, 25%, 31%, and 34% of marital satisfaction in our sample to ensure that our results were not specific to only the most dissatisfied spouses. We identified two distinct profiles of discordant marriages in all data subsets, with one profile reporting dissatisfaction in all areas, and the other reporting low marital satisfaction but close to average satisfaction with life, family, and friends. Our results emphasize that unhappy spouses are not monolithic, and that some individuals remain relatively satisfied in other life domains.

## 1 Introduction

Leo Tolstoy famously began his 1877 novel *Anna Karenina* by asserting that “All happy families are alike; each unhappy family is unhappy in its own way.” Rarely does a single quotation drive scientific exploration, but Tolstoy’s compelling hypothesis captured the interest of researchers across disciplines—from psychology to microbiology. Some have sought to challenge the first clause—are all happy families *really* alike?—while others have applied the second clause as a way to explain unique causes of dysfunction and disease (for example, see Fincham et al., 2007; Iliev and Bennis, 2023; Ma, 2020).

The present study addresses Tolstoy’s second clause: Are unhappy families (specifically, couples) all unhappy in their own way? We seek to explore whether unhappy married couples can be fruitfully divided into sub-types. Broadly speaking, evidence from relationship science seems to support this hypothesis, with numerous studies delineating varying types, causes, and consequences of marital distress (McNulty et al., 2021; Salinger et al., 2021). Although many studies have provided evidence for unique causes and outcomes of marital distress (Bradbury et al., 2000; Gottman and Silver, 2015; Tavakol et al., 2017), distressed marriages are still frequently treated as a single, uniform category by researchers and clinicians (Christensen et al., 2004). This treatment is at least partially justified by the fact that relationship scientists have identified consistent interaction and conflict styles that plague unhappy marriages, as well as predict divorce and other negative outcomes (Gottman, 1979; Raush et al., 1974). However, while some research supports the idea that unhappy couples share similarities, this may not sufficiently justify lumping them into a single category. Therefore, there is a gap in the literature—researchers, by and large, have not attempted to directly test the counter-case that there is heterogeneity among individuals in dissatisfying marriages—or, to once again quote Tolstoy, that each spouse might be “unhappy in their own way.” There are numerous forms this heterogeneity could take. For example, might some unhappily married spouses still find satisfaction in their lives and other relationships despite their unsatisfactory marriage? In contrast, could other unhappy spouses experience dissatisfaction that extends to most aspects of their lives? If such heterogeneity exists, it would not only support Tolstoy’s hypothesis about the uniqueness of unhappy families, but also carry important implications for science and practice.

In examining the heterogeneity of unhappily married couples, two prominent theoretical frameworks provide insight into the complexities of marital distress and stability. The vulnerability-stress-adaptation (VSA) model (Karney and Bradbury, 1995) highlights how enduring vulnerabilities, external stressors, and adaptive processes dynamically interact to shape relationship outcomes. This model underscores that marital stability is not solely determined by satisfaction but also by how couples adapt to stress and leverage their strengths. The brain complexity and marital behaviors (BCM) theory (Nikrahan, 2023) complements this perspective by applying principles from complexity science and neuroscience to conceptualize marriages as complex adaptive systems. According to BCM, robustness in the marital behaviors system arises from distributed interactions across various factors—such as resilience resulting from several factors including external supports and individual well-being—rather than being centrally dictated by marital satisfaction alone. These frameworks help conceptualize heterogeneity in unhappy marriages, particularly in relation to satisfaction across other domains (i.e., friendship, family, and life satisfaction). While both models emphasize adaptation to external stressors, our study does not directly test the VSA or BCM frameworks but rather uses them as a conceptual lens to interpret patterns of dissatisfaction. By integrating these perspectives, we aim to explore the extent to which some unhappily married individuals maintain satisfaction in other areas of life, shedding light on distinct patterns of discord across life domains.

In the present study, we investigate heterogeneity through the lens of a specific question: what is the “shape” of the dissatisfaction experienced by people in discordant marriages? Historically, research suggests a consequential proportion of married individuals experience low levels of marital satisfaction (see Proulx et al., 2017 for a review of marital satisfaction research). Low marital satisfaction tends to be a strong predictor of divorce (Hawkins and Booth, 2005; Lavner and Bradbury, 2010), but of course, not all unsatisfying marriages end in divorce. Research on stable unhappy marriages suggests that in some cases couples stay together despite significant distress (Heaton and Albrecht, 1991). We suggest this may be due to variation in the “distress profile” of coupled individuals. Researchers have yet to explore the possibility that people who report similar levels of marital dissatisfaction may differ substantively in their satisfaction with other life domains. To address this question, the present study used latent profile analysis (LPA) on a large preexisting cross-sectional sample of married individuals to investigate whether categorical differences exist among individuals in dissatisfying marriages. Although our study does not constitute a hard test of what has been termed Tolstoy’s “Anna Karenina Principle,” we believe the underlying sentiment—that there are substantive and meaningful differences among unhappy couples, and these differences are significant enough that unhappy couples should not be treated as a monolith—is a valuable idea. Instead, we use this sentiment as an underlying conceptual framework for exploring heterogeneity among unhappy spouses.

### 1.1 Identifying unhappy spouses

To study heterogeneity in discordant spouses, one of the first challenges involves identifying a reasonable approach for determining which relationships count as discordant. Previous work has used several approaches to define and categorize unhappy spouses and unsatisfying marriages, but some of these are unsuitable due to their arbitrariness. For example, researchers have historically relied on validated cut-points on various marital satisfaction scales, such as the Dyadic Adjustment Scale or the Marital Adjustment Test (Christensen et al., 2004; Spanier, 1976) to establish which spouses were distressed. Although clinically and diagnostically useful, cut scores are flawed because, as Beach et al. (2005) have noted, they can easily become arbitrary in some research contexts—is it appropriate, for example, for a researcher to decide that a cutoff score of “3” represents the boundary lines for misery? Cut scores, therefore do not represent a strong criterion for categorizing and understanding discordant marriages.

Over the past 20 years, researchers have begun to explore more promising methods of creating a non-arbitrary criterion of marital discord. Initially, researchers sought to establish categorical distinctions between distressed spouses and other spouses using taxometric procedures to identify whether a construct displays evidence of a latent categorical structure, or whether it is best characterized as dimensional (Beach et al., 2005; cf. Meehl and Yonce, 1996). Two cross-sectional studies, one with a small sample of first-time married couples and the other with a larger, more representative sample, used these taxometric methods to categorize married couples into separate subsets, documenting that a proportion of couples can, indeed, appropriately be viewed as distressed, with spouses becoming increasingly negative in their feelings and interactions with their partners, as expressed by low marital satisfaction scores. The same study found that a substantial number of other spouses could similarly be sorted into a non-discordant group (Beach et al., 2005). Discordant couples in this study experienced marriage in qualitatively different ways than non-discordant couples, and the structure of the study not only allowed the researchers to characterize discord, but also to estimate the size of the discordant group. In Beach et al. (2005) paper, the discordant group constituted approximately 20% of the total sample. A second study, expanding Beach et al. (2005), found that approximately 31% of a population of married couples could be categorized as experiencing marital discord (Whisman et al., 2008). These early studies suggest that a nontrivial proportion of married couples experience marital discord.

More recently, researchers have begun to explore the categorization of marital trajectories over time. For example, a recent critical review paper synthesized research on trajectories of marital satisfaction (Proulx et al., 2017). Specifically, Proulx et al. (2017) synthesized the results of 14 studies that used group-based trajectory modeling (e.g., group-based semiparametric mixture modeling and latent class growth analysis), and found that low marital satisfaction groups ranged from 22% to 34% of the included samples. Thus, extant research suggests that a sizable proportion—from one-fifth to one-third—of spouses are in relatively unsatisfactory marriages, underscoring the importance of understanding the heterogeneity among individuals experiencing low marital satisfaction.

Based on converging evidence from the literature, then, it is estimated that anywhere from the bottom 20% to 34% of all couples may be considered discordant. Unfortunately, there is not a clear, empirically derived percentage that can serve as a “cutoff” for discordance, so in the present study we opted to use an iterative procedure to test the range of possible group sizes to determine whether our results remain consistent across different cutoffs (ranging from 20% to 34%) for the discordant group. The details of this approach are discussed further below.

### 1.2 Understanding heterogeneity: are all unhappy spouses alike?

A second challenge, having identified discordant groups, is to identify an appropriate method for determining whether they are “all alike” (i.e., homogeneous) or “unhappy in their own way” (i.e., heterogeneous). Previous research focusing on distressed marriages has typically studied unhappy spouses as a homogeneous group (Christensen et al., 2004). Newer work, however, has taken a more nuanced approach, using advanced statistical techniques to model distinct patterns of marital satisfaction among spouses, and this research offers promising evidence that discordant marriages are indeed heterogeneous.

Longitudinal trajectory models, for example, suggest that individual spouses often report discrepant levels and patterns of marital satisfaction (Williamson and Lavner, 2020). One study in particular found that a substantial proportion (59.4%) of participants were not categorized in the same trajectory as their spouse, with one individual falling into a lower satisfaction trajectory than their spouse (Lavner and Bradbury, 2010). Further, the same study found that a substantial proportion of spouses in a low marital satisfaction trajectory stayed married for up to 10 years (Lavner and Bradbury, 2010). This research suggests that at the within-marriage level, partners exhibit features of heterogeneity in terms of their levels of distress and how they respond to it. However, much less is known about the between-marriage level. More clearly, there is a paucity of research that investigates the question of whether individual spouses from different discordant marriages are heterogeneous in their levels of distress, how they respond to it, and how that distress manifests across different domains of their lives.

One way to address this gap is by examining discordant profiles across multiple domains of life, rather than focusing solely on marital satisfaction. Therefore, rather than categorically distinguishing happy and unhappy couples using a single satisfaction metric, the present research sought to better understand the heterogeneity among individuals who report low marital satisfaction in terms of their satisfaction with other important domains. Specifically, we explored the dynamics between marital satisfaction, overall life satisfaction, friendship satisfaction, and family satisfaction. Although numerous studies have examined the association between each of these constructs (and, indeed, these constructs are positively associated) no studies to our knowledge have examined them simultaneously using LPA. LPA is a statistical technique used to uncover latent groups within continuous data by estimating a probability that individuals belong to a particular group (Ferguson et al., 2020). In contrast to traditional, variable-centered approaches, LPA is a person-centered analysis, and identifies groups of people within a sample based on combinations of several variables, or, indicators (Johnson, 2021). It is an ideal technique for addressing the question of heterogeneity across discordant marriages, and therefore we have chosen it as our primary analytic approach.

We argue that such an approach will address a substantive gap in relationship science. Previous research has investigated the longitudinal relationship between life satisfaction and marital satisfaction, differences in life satisfaction among married and unmarried people, and how the composition of a married person’s social network relates to their overall marital satisfaction (Birditt and Antonucci, 2007; DeMaris and Oates, 2022; Lawrence et al., 2019). These studies have furthered understanding of the dynamics between individual well-being, interpersonal relationships (outside of marriage), and marital satisfaction, but they do not explain how these variables work together. LPA methodologies are ideally suited for exploring complex combinations of multiple indicator variables by instantiating underlying patterns of variables in the form of groups (Spurk et al., 2020; Woo et al., 2018). It therefore allows researchers the ability to answer more nuanced questions. For example, can good relationships (i.e., with friends and family) buffer against an unsatisfying marriage? The present study sought to use LPA to address such questions through secondary analysis of a large preexisting dataset.

### 1.3 The present study

The approach employed in this study consisted of two components. First, we used LPA to determine whether categorically different groups exist among individuals who reported low satisfaction with their marriage—and, if so, how many such groups there are. Second, we conducted a sensitivity analysis by using LPA to re-derive the profile solution across a series of models using different cut points for discordant marriage, as defined by the literature. Doing so allowed us to determine whether the number of profiles remained the same across the range of possible “discordant marriage” groups that could be derived from a normal population of married individuals. As previously described, research has shown that between 20% and 34% of marriages are discordant (see Proulx et al., 2017 for a review). Based on this evidence, we conducted LPA on subsets of our sample that represented the lowest 20% of marital satisfaction, the lowest 34% of marital satisfaction, along with several intermediary points (22.5%, 25%, and 31%). This approach allowed us to examine the robustness of our findings—that is, whether the best-fitting profile solution varied for subgroups of spouses who reported different levels of marital satisfaction.

#### 1.3.1 Profile indicators and covariates

A final consideration, then, is how to characterize distress in discordant marriages. To do this we suggest that a useful approach is to expand the conception of distress to encompass both the “external supports” that exist in a married person’s life as well as their global evaluations of their life. Regarding external supports, researchers have speculated that some unhappy couples see marriage as a backdrop and focus their energy on their friends, outside activities, and children (Huston et al., 2001). Accordingly, satisfaction in domains outside of one’s marriage may offset the negative impact of low marital satisfaction. Specifically, good relationships with friends and family may satisfy individuals’ fundamental need for connection (cf. Baumeister and Leary, 1995). Indeed, a substantial amount of research exists to support the conclusion that satisfying relationships with family members (e.g., Birditt and Antonucci, 2007; Fuller-Iglesias et al., 2015) and friends (e.g., Antonucci et al., 2001; Kaufman et al., 2022c) contribute in substantive ways to well-being. This is true at the level of specific non-marriage relationships, such as best-friendship; having a best friend has been shown to predict greater individual happiness (Demir et al., 2007), as well as better mental health from adolescence to young adulthood (Narr et al., 2019). Married women with close friendships also report fewer depressive symptoms and higher life satisfaction (Antonucci et al., 2001). Additionally, recent work has demonstrated that this is true at the level of social relationships in general (i.e., including, but not limited to marriage; see Holt-Lunstad et al., 2017). And there is also evidence that the general categories of friends and family play a role; a recent LPA of unpartnered, single adults found that the happiest profile was characterized by high satisfaction with both friends and family, whereas those in the least happy profile were also least satisfied with these close relationships (Walsh et al., 2022).

Regarding relationships, there is evidence that overall life satisfaction is influenced by variables that transcend the effects of specific domains such as marriage, friendship, or family. In the context of this study, it makes sense to include a measure of life satisfaction for two reasons. First, life satisfaction is a global assessment that may serve as a broad indicator that a person is deriving support from additional sources beyond friends and family. If a person has an unhappy marriage, for example, a high life satisfaction score may indicate they are drawing satisfaction from other external domains (e.g., their job, community, and/or religion) that were not specifically assessed. Life satisfaction is therefore a good general index of happiness external to marriage. Second, individuals tend to have a stable happiness “set point” that contains a hereditary component and varies from person to person (Lyubomirsky et al., 2005b). Therefore, including a measure of global life satisfaction in LPA helps index the degree to which a person has a temperament that naturally disposes them to maintaining their happiness despite a discordant marriage.

To further enrich the understanding of heterogeneity among unhappily married individuals, we selected several covariates based on their well-established associations with marital satisfaction and well-being. Neuroticism, a key personality trait, is consistently linked to reduced well-being and negative relationship outcomes, including heightened conflict and lower marital satisfaction, making it an important factor to consider (Karney and Bradbury, 1997; Ozer and Benet-Martínez, 2006). Loneliness was included as it reflects the broader social disconnection that can exacerbate marital distress and differentiate between those who find support in other relationships versus those who feel isolated (Russell, 1996). Subjective evaluations of global health are associated with better life and marital satisfaction, as well as better adherence to medical recommendations and fewer physical symptoms (Lyubomirsky et al., 2005a; Robles et al., 2014). Self-esteem was chosen for its role in shaping individuals’ perceptions of their relationships and their ability to navigate interpersonal challenges, as well as its strong links to well-being (Lyubomirsky et al., 2006; Rosenberg, 1965). Similarly, perceived stress was included as it captures an individual’s broader emotional state and their capacity to manage stressors, both of which can influence marital dynamics and mental health (Cohen et al., 1983). Finally, best friend status (i.e., having or not having a best friend) was included as strong friendships may act as a buffer against marital dissatisfaction and provide vital emotional support (Antonucci et al., 2001; Demir and Özdemir, 2010). Together, these covariates allow for a more nuanced exploration of the emergent profiles, highlighting how individual traits and external social resources contribute to the heterogeneity among unhappily married spouses.

In the present study, then, we used friendship satisfaction, family satisfaction, and life satisfaction as our primary indicators for creating latent profiles among unhappily married individuals. We hypothesized that each subset of the sample (i.e., the lowest 20%, 22.5%, 25%, 31%, and 34% of marital satisfaction) would contain more than one class with meaningful and distinct profiles, and that these profiles would remain stable (i.e., not be significantly different) among the percentile cut points. In other words, we hypothesized that there exist categorically different types of unhappily married individuals, characterized by differences in satisfaction with other interpersonal relationships and individual well-being. We also expected these profiles would exhibit significant differences on the selected covariates (neuroticism, loneliness, self-esteem, perceived stress, and best friend status).

## 2 Materials and methods

### 2.1 Data source

The present study is a secondary analysis of data collected for a prior study (see Study 2 of Kaufman et al., 2022b). This secondary data analysis was not preregistered. A nationally representative sample of participants were invited to participate in a 20-min online survey in exchange for cash compensation or its equivalent in reward points or discounts. Participants were recruited through Dynata,^1^ a global data insights and research platform. The study sample recruitment was based on a stratified approach designed to yield demographics approximating national distributions based on data from the 2010 U.S. Census and the 2018 American Community Survey, with sample distribution targets based on age, gender, race, and income (see Table 1 for demographic information). Most demographic parameters in the sample were within 1% point of their corresponding national targets.

**TABLE 1:** Demographic characteristics by subset.

	All married participants	Lowest 34% of marital satisfaction	Lowest 31% of marital satisfaction	Lowest 25% of marital satisfaction	Lowest 22.5% of marital satisfaction	Lowest 20% of marital satisfaction
**Sex**						
Male	528 (49.35%)	169 (46.43%)	151 (45.48%)	109 (42.25%)	101 (41.91%)	87 (41.04%)
Female	542 (50.65%)	195 (53.57%)	181 (54.52%)	149 (57.75%)	140 (58.09%)	125 (58.96%)
**Education**						
<High school	13 (1.21%)	4 (1.10%)	3 (0.90%)	3 (1.16%)	3 (1.24%)	3 (1.42%)
High school	118 (11.03%)	43 (11.81%)	39 (11.75%)	32 (12.40%)	31 (12.86%)	28 (13.21%)
Some college	209 (19.53%)	72 (19.78%)	67 (20.18%)	54 (20.93%)	49 (20.33%)	42 (19.81%)
College	454 (42.43%)	149 (40.93%)	134 (40.36%)	109 (42.25%)	103 (42.74%)	90 (42.45%)
Postgraduate	274 (25.61%)	95 (26.10%)	88 (26.51%)	59 (22.87%)	54 (22.41%)	49 (23.11%)
Prefer not to answer	2 (0.19%)	1 (0.27%)	1 (0.30%)	1 (0.39%)	1 (0.41%)	0 (0%)
**Income**						
Less than $30,000	64 (5.98%)	29 (7.97%)	26 (7.83%)	20 (7.75%)	19 (7.88%)	16 (7.55%)
$30,000–$49,999	102 (9.53%)	45 (12.36%)	44 (13.25%)	37 (14.34%)	35 (14.52%)	32 (15.09%)
$50,000–$74,999	159 (14.86%)	47 (12.91%)	45 (13.55%)	36 (13.95%)	32 (13.28%)	26 (12.26%)
$75,000–$99,999	198 (18.50%)	64 (17.58%)	56 (16.87%)	41 (15.89%)	38 (15.77%)	33 (15.57%)
$100,000–$149,999	249 (23.27%)	76 (20.88%)	67 (20.18%)	52 (20.16%)	48 (19.92%)	43 (20.28%)
$150,000 or greater	298 (27.85%)	103 (28.30%)	94 (28.31%)	72 (27.91%)	69 (28.63%)	62 (29.25%)
Age	49.3 (13.9)	49.5 (12.8)	49.7 (12.8)	49.7 (12.8)	49.7 (12.9)	49.6 (13.0)
**Race/ethnicity**						
White or Caucasian	737 (68.88%)	248 (68.13%)	223 (67.17%)	176 (68.22%)	167 (69.29%)	142 (66.98%)
Black or African American	94 (8.79%)	30 (8.24%)	30 (9.04%)	20 (7.75%)	18 (7.47%)	16 (7.55%)
Hispanic and/or Latino	165 (15.42%)	62 (17.03%)	57 (17.17%)	46 (17.83%)	41 (17.01%)	40 (18.87%)
American Indian/Alaska Native	45 (4.21%)	19 (5.22%)	18 (5.42%)	13 (5.04%)	12 (4.98%)	11 (5.19%)
Asian	8 (0.75%)	2 (0.55%)	2 (0.60%)	2 (0.78%)	2 (0.83%)	2 (0.94%)
Native Hawaiian/Pacific Islander	1 (0.09%)	1 (0.27%)	1 (0.30%)	1 (0.39%)	1 (0.41%)	1 (0.47%)
Other	20 (1.87%)	2 (0.55%)	1 (0.30%)	0 (0%)	0 (0%)	0 (0%)

To maximize data integrity, five engagement checks were randomly included throughout the survey to verify that participants were paying attention. In total, 3,699 participants completed the survey; of those, the 2,000 participants who passed every engagement check comprised the final sample of usable data. In the final sample of 2,000 participants, 1,070 (53.5%) were married and 930 (46.5%) were unmarried (widowed, divorced, separated, or never married). After excluding unmarried participants, the present analysis includes data from 1,070 married spouses (not dyads) from the final sample. All procedures for data collection were submitted to and received approval from our university’s Institutional Review Board.

### 2.2 Sample characteristics

Demographic characteristics for the entire sample of married participants (*N* = 1,070) are presented in Table 1.

### 2.3 Measures

#### 2.3.1 Outcome variable: marital satisfaction

Marital satisfaction was measured using a sum composite of the 16-item Couples Satisfaction Index (Funk and Rogge, 2007). Participants rated items such as “My relationship makes me happy” and “I really feel like part of a team with my partner” on a scale from 0 (*not at all true*) to 5 (*completely true*). Other items were rated on different scales (e.g., “Please indicate the degree of happiness, all things considered, of your relationship” was rated on a scale from 0 *extremely unhappy* to 6 *perfect*). Cronbach’s α ranged from 0.95 to 0.98 (see Supplementary Table 1 for Cronbach’s α for the entire dataset and by subset for each measure).

#### 2.3.2 Indicator variables

##### 2.3.2.1 Life satisfaction

Life satisfaction was measured using a sum composite of the 5-item Satisfaction With Life Scale (SWLS; Diener et al., 1985; e.g., “In most ways my life is close to my ideal”) rated from 1 (*strongly disagree*) to 7 (*strongly disagree*) and the 8-item Personal Wellbeing Index (PWI; Lau et al., 2005; e.g., “How satisfied are you with your standard of living?”) rating from 0 (*no satisfaction at all*) to 10 (*completely satisfied*). We combined these two scales based on previous work showing the two scales are highly correlated, highly reliable when combined, and both belong to a higher-order subjective well-being latent construct (Kaufman et al., 2022a; Kaufman et al., 2022c; Walsh et al., 2022). Cronbach’s α ranged from 0.93 to 0.94 for the well-being composite across sample subsets (i.e., the lowest 20%, 22.5%, 25%, 31%, and 34% of marital satisfaction).

##### 2.3.2.2 Family satisfaction

Family satisfaction was assessed using the 10-item Family Satisfaction Scale (Olson, 1985). Participants rated each item on a 6-point scale from 1 (*very dissatisfied*) to 5 (*extremely satisfied*). Example items include “The degree of closeness between family members” and “The quality of communication between family members.” Cronbach’s α ranged from 0.93 to 0.96.

##### 2.3.2.3 Friendship satisfaction

Friendship satisfaction was measured using 14 items from the Friendship Network Satisfaction Scale (Kaufman et al., 2022b). Participants rated their agreement with items such as “My friends celebrate my good news” on a scale on a 6-point from 0 (*not at all agree)* to 5 (*completely agree*). Cronbach’s α was 0.96 for all subsets.

#### 2.3.3 Covariates

In addition to the above outcome and indicator variables, we also assessed the below covariates. In LPA, covariates are antecedent variables that theoretically have no effect on the formation of the profiles. However, examining such covariates may further validate and differentiate the resulting profiles.

##### 2.3.3.1 Neuroticism

Neuroticism was measured using the neuroticism subscale (all questions) of the Eysenck Personality Questionnaire (Eysenck and Eysenck, 1993). Participants rated items such as “Does your mood often go up and down?” from 1 (*yes*) to 0 (*no*). Cronbach’s α was 0.93 for all subsets.

##### 2.3.3.2 Loneliness

Loneliness was measured using 8 items of the UCLA Loneliness Scale (Russell, 1996). Participants rated items such as “I feel left out” on a scale from 1 (*never*) to 4 (*often*). Cronbach’s α ranged from 0.84 to 0.87.

##### 2.3.3.3 Subjective health

Subjective health was measured with a single item from the Patient-Reported Outcomes Measurement Information System (PROMIS) Global Health Scale (Hays et al., 2015). Participants rated their health (“In general, would you say your health is:”) on a scale from 1 (*excellent*) to 5 (*poor*). This item was reverse-coded so that higher numbers reflect better self-reported health.

##### 2.3.3.4 Self-esteem

Self-esteem was measured using the 10-item Rosenberg Self-Esteem Scale (Rosenberg, 1965). Participants responded to items such as “I feel that I have a number of good qualities” on a scale from 1 (*strongly disagree*) to 4 (*strongly agree*). Cronbach’s α ranged from 0.89 to 0.91.

##### 2.3.3.5 Perceived stress

Perceived stress was measured using the 4-item Perceived Stress Scale (PSS; Cohen et al., 1983). Participants indicated the frequency of items such as “In the last month, how often have you felt difficulties were piling up so high that you could not overcome them?” on a scale from 0 (*never*) to 4 (*very often*). Cronbach’s α ranged from 0.73 to 0.79.

##### 2.3.3.6 Best friend status

Best friend status was assessed with a single face-valid question (“Do you have a best friend”; Kaufman et al., 2022b). Participants had three option choices: (1) *Yes*; (2) *No, I have multiple friends who I am very close with*; or (3) *No, I do not have anyone to call a best friend*.

##### 2.3.3.7 Demographic characteristics

Participants also completed demographic information about their age, gender, education, and income, which were also included as covariates.

## 3 Analytic approach

We conducted LPA to identify latent homogeneous subgroups using friendship satisfaction, family satisfaction, and life satisfaction as indicator variables, and marital satisfaction as the outcome variable. LPA were conducted using Mplus (Version 8.1; Muthén and Muthén, 1998–2018) and the Mplus Automation R package (Hallquist and Wiley, 2018). All variables were continuous and *z*-scored based on the entire sample of married participants (*M* = 0; SD = 1) before analysis.

We performed LPA on five subsets of our data, each varying in their level of self-reported marital satisfaction. Specifically, we performed LPA among participants with marital satisfaction in the lowest 20% (*n* = 212), 22.5% (*n* = 241), 25% (*n* = 258), 31% (*n* = 332), and 34% (*n* = 364) of the full married sample (*N* = 1,070). We used model fit statistics including −2 Log-Likelihood (−2LL), Akaike information criterion (AIC), Bayesian information criterion (BIC), sample size-adjusted Bayesian information criterion (aBIC), Vuong-Lo-Mendell-Rubin Likelihood Ratio Test (VLMRT), and Lo-Mendell-Rubin Test (LMRT) to determine the optimal number of latent profiles at each percentile cutoff point. A better model fit was indicated by lower values of −2LL, AIC, BIC, and aBIC (Nylund et al., 2007). Once we identified an optimal solution for each cut, individuals were assigned to specific profiles based on the greatest probability of group membership. Next, the most likely latent profile membership was accounted for measurement error in profile assignment by using logits for the classification probabilities to fix measurement parameters, resulting in a final model being estimated. Finally, we inspected differences among the profiles on the primary outcome (marital satisfaction) and covariates. Specifically, we compared individuals in terms of their self-reported health, neuroticism, loneliness, and self-esteem, and perceived stress. We also examined profile differences on demographic characteristics, such as age, gender, education, and income. It is important to note that marital satisfaction is initially used to define low levels of marital satisfaction consistent with thresholds previously discussed. We then use marital satisfaction as a primary outcome to compare means between emergent profiles. These analyses allowed us to validate and contextualize the profiles identified in our analyses, and to better understand the extent to which profile membership was associated with differences in psychological and social functioning. To this end, we used manual three-step auxiliary BCH approach (see Table 2) for continuous variables and DCAT approach (see Supplementary Table 5) for categorical variables to test group differences by applying Wald χ^2^ tests (Asparouhov and Muthén, 2014).

**TABLE 2:** Relationships between profile membership, outcome, and covariates.

	Outcome	Covariates				
	**Marital satisfaction**	**Neuroticism**	**Perceived stress**	**Loneliness**	**Subjective health**	**Self-esteem**
	***M* (SE)**	***M* (SE)**	***M* (SE)**	***M* (SE)**	***M* (SE)**	***M* (SE)**
**20% subset**						
Profile 1	−1.97 (0.09)	1.29 (0.12)	1.3 (0.1)	1.07 (0.1)	−0.87 (0.11)	−1.30 (0.12)
Profile 2	−1.34 (0.07)	−0.04 (0.11)	0.01 (0.1)	0.17 (0.1)	−0.11 (0.11)	0.08 (0.1)
Wald χ^2^	25.483***	58.249***	71.773***	33.375***	19.97***	71.356***
**22.5% subset**						
Profile 1	−1.84 (0.09)	1.23 (0.11)	1.28 (0.09)	1.03 (0.1)	−0.78 (0.1)	−1.27 (0.11)
Profile 2	−1.25 (0.07)	−0.05 (0.1)	0.01 (0.09)	0.18 (0.09)	−0.13 (0.1)	0.04 (0.09)
Wald χ^2^	23.886***	66.43***	88.379***	36.428***	17.095***	74.124***
**25% subset**						
Profile 1	−1.81 (0.09)	1.25 (0.11)	1.28 (0.09)	1.03 (0.1)	−0.80 (0.1)	−1.27 (0.11)
Profile 2	−1.18 (0.07)	−0.04 (0.09)	0.07 (0.09)	0.18 (0.09)	−0.13 (0.09)	0.01 (0.09)
Wald χ^2^	27.78***	68.251***	79.758***	35.807***	19.157***	71.146***
**31% subset**						
Profile 1	−1.76 (0.06)	1.29 (0.08)	1.28 (0.07)	1.07 (0.07)	−0.86 (0.08)	−1.34 (0.07)
Profile 2	−0.9 (0.1)	−0.07 (0.12)	0.03 (0.1)	0.17 (0.11)	−0.13 (0.11)	0.02 (0.11)
Wald χ^2^	46.628***	80.243***	88.244***	40.8***	24.977***	88.472***
**34% subset**						
Profile 1	−1.72 (0.1)	1.23 (0.11)	1.23 (0.09)	1.06 (0.1)	−0.80 (0.11)	−1.29 (0.11)
Profile 2	−0.79 (0.05)	−0.06 (0.07)	0.06 (0.07)	0.14 (0.07)	−0.09 (0.07)	−0.03 (0.07)
Wald χ^2^	56.804***	75.118***	85.338***	45.488***	24.392***	80.104***

Standardized using *z*-scores (full sample *M* = 0, SD = 1). Profiles were constructed based on the three indicator variables (life satisfaction, friendship satisfaction, and family satisfaction). Profile 1: globally dissatisfied (very low friend, family, and life satisfaction). Profile 2: partially satisfied (average friendship satisfaction, slightly low life satisfaction, and low family satisfaction).

***p < 0.001.

## 4 Results

Across all discordant subsets, model fit indices consistently indicated that the 2-profile solution was optimal. We then examined indicator (i.e., friendship satisfaction, family satisfaction, and life satisfaction) *z*-scores to label, describe, and understand the profiles. Finally, we compared emergent profiles on auxiliary variables (i.e., outcome and covariates). The details for each of these analyses are described below.

### 4.1 Latent profile analysis

Using LPA, we identified heterogeneous groups of individuals with low marital satisfaction in all subsets of our data (i.e., participants in the lowest 20%, 22.5%, 25%, 31%, and 34% of marital satisfaction in the full sample of married individuals). See Supplementary material for LPA model fit indices (including −2LL, AIC, BIC, aBIC, VLMRT, and LMRT) for each solution in each subset (see Supplementary Table 2). We estimated 1-, 2-, and 3-profile solutions (except for the 34% model, in which case we assessed up to a 4-profile solution). The average probabilities for most likely class membership were between 0.85 and 0.90 for each model (see Supplementary Table 3). Based on these values, we selected the two-class model for each of our data subsets, since this solution was parsimonious, easily identified, fit best based on the BIC values, and its parameter estimates presented a solution with a logical substantive solution. We note, however, that two of the percentage solutions had statistically significant findings based on the LMRT test for the three-class solutions, but in each of these cases, one of the classes was very small (i.e., 3 and 18 people).

### 4.2 Describing the profiles

**FIGURE 1:**
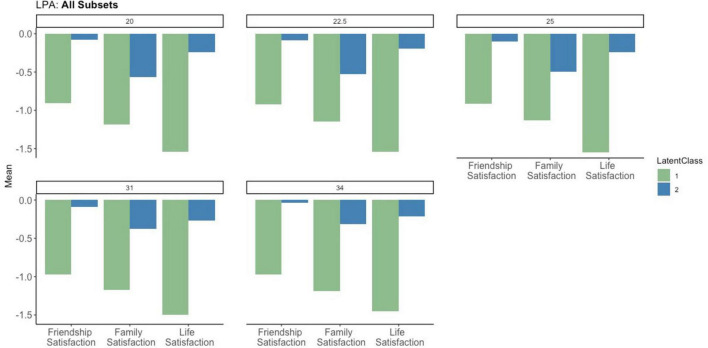
Latent profile analysis indicator patterns for all subsets.

In all subsets, we identified two latent profiles as the best-fitting solution in our analysis. Here, we describe the differences in profiles first by their indicators. Because all variables were standardized (see Supplementary Table 4 for unstandardized means for each variable), we describe the means for each profile in terms of Cohen’s (1992) effect size range thresholds whereby *d* = 0.20 represents a small effect, *d* = 0.50 represents a medium effect, and *d* = 0.80 represents a large effect. Thus, we categorize *z*-score means of ±0 to ±0.20 as “average,” means of ±0.20 to ±0.50 as “slightly high” or “slightly low,” means of ±0.50 to ±0.80 as “high” or “low,” and means ± 0.80 and above/below as “very high” or “very low.” The patterns of indicator variables for each profile were remarkably similar among all subsets (see Figure 1 for a side-by-side comparison of all subsets). For illustrative purposes, we include graphs for each subset in Supplementary material. Finally, because specific numeric results differed slightly among subsets, we describe high-level patterns for each profile below and include numeric results in Table 2.

#### 4.2.1 Profile 1: globally dissatisfied (very low friend, family, and life satisfaction)

Participants in profile 1 had uniformly unfavorable indicator patterns. Specifically, these participants were below average on all indicators, with very low satisfaction with their friends (means ranged from −0.90 to −0.97), family (means ranged from −1.13 to −1.19), and life (means ranged from −1.45 to −1.54 SD below average).

#### 4.2.2 Profile 2: partially satisfied (average friendship satisfaction, slightly low life satisfaction, and low family satisfaction)

Participants in profile 2 had more favorable indicator patterns than those in profile 1. These participants had average friendship satisfaction (means ranged from −0.08 to −0.11) and slightly low life satisfaction (means ranged from −0.20 to −0.27). Family satisfaction was the most unfavorable indicator for those in profile 2, with means ranging from slightly low to low (−0.32 to −0.57). This could be due to the fact that family satisfaction is partially confounded with marital satisfaction, which we are not able to disentangle further in the present study. Notably, no indicator was over one standard deviation below the mean of all married participants. This suggests that although participants in both profiles report low marital satisfaction (i.e., among those who reported the lowest 20%-34% of marital satisfaction), this dissatisfaction may be relatively domain-specific for those in profile 2, who report relatively average friendship and life satisfaction.

### 4.3 BCH and DCAT analyses

Finally, we used the manual three-step auxiliary BCH approach for continuous variables and DCAT approach for dichotomous variables to test group differences among the profiles.

#### 4.3.1 Outcome: marital satisfaction

Profile membership was significantly associated with marital satisfaction in all subsets (χ^2^ ranged from 23.88 to 56.80, all *p*s < 0.001). Overall, although marital satisfaction was very low among participants in both profiles (<−0.80), participants in profile 1 had significantly lower marital satisfaction than those in profile 2. Participants in profile 1 reported mean levels of marital satisfaction ranging from −1.72 to −1.97, whereas those in profile 2 ranged from −0.79 to −1.34 (see Table 2 for exact means for each profile). For both profiles, marital satisfaction was less negative in each consecutive subset, which makes sense given that, by definition, the subsets vary by level of marital satisfaction.

In terms of profile size, at low levels of marital satisfaction (i.e., the lowest 20%, 22.5%, and 25%), the profiles were split almost equally (see Table 3 for exact profile counts for each subset). Notably, the likelihood of membership in profile 2 (i.e., the profile with relatively more favorable indicator patterns) increased with martial satisfaction. That is, among the subsets including the lowest 31% and 34% of marital satisfaction, profile 2 represented about 60% (62% in the lowest 31% subset, 63% in the 34% subset) of the sample, compared to about 50% in the least satisfied subsets (from 48% to 52% among those with the lowest 20%-25% of marital satisfaction). This could be evidence of an upper limit of defining discordant marriages as between 20% and 30%, which aligns with prior literature (Proulx et al., 2017).

**TABLE 3:** Profile membership counts by subset.

		Profile 1		Profile 2		Change		
**Subset**	**Subset** ***n***	**Profile** ***n***	**Percent of subset**	**Profile** ***n***	**Percent of subset**	**Total *n* increase**	**% added to profile 1**	**% added to profile 2**
Lowest 20%	212	111	52.36%	101	47.64%	–	–	–
Lowest 22.5%	241	120	49.79%	121	50.21%	29	31.03%	68.97%
Lowest 25%	258	124	48.06%	134	51.94%	17	23.53%	76.47%
Lowest 31%	332	128	38.55%	204	61.45%	74	5.41%	94.59%
Lowest 34%	364	134	36.81%	230	63.19%	32	18.75%	81.25%

Total *n* increase reflects the number of individuals added to each subset, and the “% added” columns reflect the proportion of individuals added to each profile. Profile 1: globally dissatisfied (very low friend, family, and life satisfaction). Profile 2: partially satisfied (average friendship satisfaction, slightly low life satisfaction, and low family satisfaction).

#### 4.3.2 Covariates of profile membership

**FIGURE 2:**
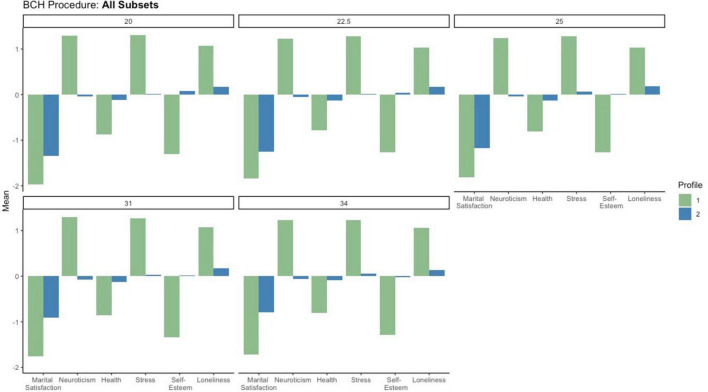
BCH procedure for all subsets.

Like the results of our latent profile analyses, the relationships between profile membership and covariates were highly similar among all subsets (see Figure 2 for a side-by-side comparison of continuous covariates for all subsets). Below we discuss each covariate.

##### 4.3.2.1 Neuroticism

Neuroticism was significantly associated with profile membership in all subsets (χ^2^ ranged from 58.25 to 80.24, all *p*s < 0.001). In line with the indicator patterns, participants in profile 1 were more neurotic than those in profile 2. Further, participants in profile 1 were above average in neuroticism (mean neuroticism ranged from 1.23 to 1.29 SD above average) whereas those in profile 2 reported average neuroticism (mean neuroticism ranged from 0.04 to 0.07 SD below average).

##### 4.3.2.2 Loneliness

Loneliness was significantly associated with profile membership in all subsets (χ^2^ ranged from 33.38 to 45.49, all *p*s < 0.001). Participants in profile 1 reported experiencing more loneliness than those in profile 2. Specifically, participants in profile 1 reported experiencing very high levels of loneliness (mean loneliness ranged from 1.03 to 1.07 SD above average) whereas those in profile 2 reported slightly higher than average levels of loneliness (mean loneliness ranged from 0.14 to 0.18).

##### 4.3.2.3 Subjective health

Subjective health was significantly associated with profile membership in all subsets (χ^2^ ranged from 17.10 to 24.98, all *p*s < 0.001). Participants in profile 1 reported poorer overall health than those in profile 2. Specifically, participants in profile 1 reported experiencing low levels of self-reported physical health (means ranged from 0.78 to 0.87 SD below average) whereas those in profile 2 reported average health (means ranged from −0.09 to −0.13).

##### 4.3.2.4 Self-esteem

Self-esteem was significantly associated with profile membership in all subsets (χ^2^ ranged from 71.15 to 88.47, all *p*s < 0.001). In line with the indicator patterns, participants in profile 1 reported lower self-esteem than those in profile 2. Specifically, participants in profile 1 were below average in self-esteem (mean self-esteem ranged from 1.27 to 1.34 SD below average) whereas those in profile 2 reported average self-esteem (mean self-esteem −0.03 to 0.08).

##### 4.3.2.5 Perceived stress

Perceived stress was significantly associated with profile membership in all subsets (χ^2^ ranged from 71.77 to 88.38, all *p*s < 0.001). Participants in profile 1 reported experiencing more stress than those in profile 2. Specifically, participants in profile 1 reported experiencing very high levels of stress (mean stress ranged from 1.23 to 1.30 SD above average) whereas those in profile 2 reported slightly average levels of stress (mean stress ranged from 0.01 to 0.07).

##### 4.3.2.6 Best friend status

Best friend status was significantly associated with profile membership in all subsets. Specifically, participants in profile 1 were more likely to report that they did not have a best friend compared to those in profile 2, which aligns with profile differences in the indicator variables (i.e., friendship satisfaction) and auxiliary variables (i.e., loneliness).

##### 4.3.2.7 Demographic characteristics

Profiles did not significantly differ by age or gender (all *p*s > 0.05).

Education was significantly associated with profile membership, but the pattern of results was mixed among our five subsets. The association between education and profile membership was marginally significant for the 20% and 22.5% subsets, and statistically significant for the 25%, 31%, and 34% subsets. Across these three subsets, participants in profile 2 were more likely to have a postgraduate education than those in profile 1.

Income was significantly associated with profile membership among participants in all five subsets, although these differences tended to be small, and limited to differences in extreme income categories. Across subsets, participants in profile 1 were more likely to report incomes of less than $30,000 per year than those in profile 2, and those in profile 2 were more likely to report incomes of $150,000 or more than those in profile 1.

## 5 Discussion

Previous research suggests that a sizable proportion of married couples report low marital satisfaction (Proulx et al., 2017). Historically these discordant marriages have been treated as a monolith, but there is reason to care about variability within this group. Marital dissatisfaction is associated with negative health outcomes for the individuals involved in the marriage (e.g., Lev-Ari et al., 2021; Shrout et al., 2021) as well as the fate of the marriage itself (Whisman and Collazos, 2023). Given this, we think it is important to ask whether these negative outcomes are distributed evenly or if some discordant couples are more “at risk” than others. To this end, we used a person-centered approach (LPA) to determine whether distinct profiles of unhappily married individuals could be identified in a large existing dataset. Since scholars tend to disagree on the boundary line for “discordant marriages,” we built a robustness check into our research by replicating our results across five subsets of the data, including participants who reported the lowest 20%, 22.5%, 25%, 31%, and 34% of marital satisfaction.

So, what did this approach show? Our hypothesis that there would be distinct profiles was supported within every subset tested. But the more interesting finding is the conceptual differences between those subgroups; using our indicator variables (life satisfaction, family satisfaction, and friendship satisfaction), our best-fitting solution consisted of two latent profiles exhibiting shape differences in our indicators, which replicated across all tested subsets.

The shape of the first globally dissatisfied profile is consistent with a pattern of uniform distress: members of that profile were very unhappy in their marriages and this unhappiness was mirrored in every other indicator and covariate in our analysis. Members of the first profile reported very low satisfaction with their friends, families, and life in general. In terms of covariates, they were higher in neuroticism, stress, and loneliness, plus lower in subjective health and self-esteem. While they were especially maritally dissatisfied, there was no area where their dissatisfaction did not reach.

In contrast, the shape of the second partially satisfied profile is consistent with a pattern of buffered distress, where negativity in marriage is more isolated to that specific area. Members of the second profile were moderately unhappy with their marriages but their unhappiness appeared largely contained. In terms of profile indicators, they were slightly lower than average on family and life satisfaction, which both overlap conceptually with marital satisfaction (i.e., spouses are a type of family member, and marital satisfaction has a strong positive correlation with life satisfaction; Carr et al., 2014). In contrast, the least related indicator, friendship satisfaction, was close to the average of the full sample—suggesting friendship may partially offset the negative effects of a dissatisfying marriage. Participants in this profile were also close to average on our covariates—their greatest deviations from the mean of the full sample were in loneliness and subjective health.

The story told by these groups is simple but has substantive implications. There is one group of people in discordant marriages whose unhappiness extends outside of their marriage into non-marital domains, and a second group whose unhappiness appears to be more isolated to their marriage. Since we tested multiple subsets of data, from the lowest 20% to 34% of marital satisfaction, we can also discuss the relative prevalence of these two groups. In the subset of our sample that scores in the lowest 34%, approximately one-third of the subset (36.81%) were globally dissatisfied. In the subset that scored in the lowest 20% of marital dissatisfaction, approximately half (52.36%) were globally dissatisfied. This suggests that even at higher levels of marital discord there is still a substantial divide between those who have more successfully isolated their dissatisfaction and those who have not.

The natural follow up question is, why is this the case? Our findings are consistent with a range of possible explanations. Two possible explanations are causal, and they may be theoretically considered but obviously not confirmed from our cross-sectional data. One possible explanation is that marital dissatisfaction radiates into other areas of life when a critical threshold of marital dissatisfaction has been reached. A second possible explanation is that marital dissatisfaction is manageable if other areas of life are doing well—but increases rapidly when they are not. However, both of these explanations would (presumably) be visible in our data as we move from the less restrictive 34% subset to the more restrictive 20% subset. Average levels of marital dissatisfaction do indeed increase (as would be expected) moving from the 34% subset to the 20% subset. If the two explanations offered above were consistent with the data, then we would expect the relevant variables to covary with marital dissatisfaction. However, they do not; the average level of the covariates (neuroticism, perceived stress, loneliness, subjective health, and self-esteem) remain largely unchanged across subsets. This is also true of two of the three indicators, friendship satisfaction and life satisfaction. Thus, this general absence of covariation between marital satisfaction and indicators/covariates across subsets speaks against a causal relationship.

This suggests that whatever variable produces these effects may do so by determining which profile individuals wind up in. There are a few additional possible explanations that are consistent with this that draw upon the VSA model first proposed by Karney and Bradbury (1995). Per the VSA, each spouse’s characteristics, or, vulnerabilities, contribute to the satisfaction with and quality of a marriage by influencing the couple’s ability to adapt to stressors within and outside of the relationship. We can think of a few candidate variables which may influence this vulnerability.

One possibility is demographic indicators of life security. Our analyses suggest that those in the globally dissatisfied profile tend to be lower in several key demographic indicators of resources than those in the partially satisfied profile. Overall, those in the globally dissatisfied profile tend to have slightly lower income and tend to be marginally less educated—although given the modest size of the differences it appears unlikely that these variables alone may explain the gap between profiles. The groups also differ in terms of external social support—those in the globally dissatisfied profile are, in all subsets, at least twice as likely to say that they have no best friend as their peers in the partially satisfied profile. This implies they may lack other reliable social relationships to turn to.

A second possibility is that individual-level psychopathology may play a role; given the stark differences between the two profiles in terms of negative covariates (neuroticism, self-esteem, stress, self-reported health, and loneliness) it is possible that such effects may be produced by a variable that the covariates share in common, such as depression. There is a strong argument to be made for this: meta-analytic research has shown an association between depression and a pessimistic attribution style, marked by a tendency to attribute situational problems to causes that are global, immutable, and internal, as well as by a belief that problems in one area have radiating negative implications for other areas (Haehner et al., 2024). If that is the case, the pessimistic attribution style associated with depression could take a situational problem (like a discordant marriage) and elevate it to a global threat to well-being.

Alternately, the explanation could lie in dyad-level variables that characterize the marriage, such as power distribution—that is, is power shared in the relationship in an egalitarian fashion, or distributed asymmetrically? And if asymmetric, which partner is more powerful? Research suggests that the experience of subjective power in relationships influences several outcomes related to the processing of negative emotion including the ability to repress negativity to focus on desired goals (Cho and Keltner, 2020), which may also explain the patterns seen in our data. More research is necessary to understand what relationship these individual and dyad-level variables have to one’s ability to buffer against a discordant marriage, but this is a potentially fruitful avenue of research.

Notably, our findings suggest that some unhappily married individuals experience dissatisfaction that extends across multiple life domains, whereas others maintain relative satisfaction in non-marital areas such as friendships and life satisfaction. Initially, we framed this as a research question, exploring whether satisfaction in external domains might buffer the negative effects of marital dissatisfaction. However, our findings suggest a more nuanced interpretation—while individuals in the partially satisfied profile report greater satisfaction in some domains, these differences may reflect pre-existing individual traits rather than a direct buffering mechanism. As such, we present the buffering effect as an interpretative framework rather than a formal hypothesis, and we caution against assuming a causal relationship. Future research should directly examine whether satisfaction in external domains actively mitigates marital distress, ideally using longitudinal or experimental designs to assess the directionality of these associations.

Overall, our results have several implications for research and practice. First, our results support the notion that unhappily married individuals comprise at least two distinct groups. This suggests that discordant marriages may vary in terms of overall risk for negative outcomes. While some may be truly dysfunctional, others may be stable and may even improve without intervention (Beach et al., 2005). If that is the case, the presence of a globally dissatisfied and a partially satisfied distress profile would imply differential approaches to clinical intervention and treatment. For example, a person in the globally dissatisfied profile may benefit from both individual and couples’ counseling to improve satisfaction across other life domains, rather than focusing exclusively on their marriage. As precursor to this sort of clinical application, however, research must first be conducted establishing a link between these profiles and negative outcomes like divorce and pathology. Such precursory work should also provide empirical evidence for plausible mechanisms.

### 5.1 Limitations and future directions

The present research has several limitations that are important to acknowledge. First, the present study is a secondary analysis of a large, existing dataset. Because of this, some relevant variables were not measured and could not be included in our analyses. For example, we did not have access to a measure of marriage/relationship length, which is a key covariate to include in future research. Future research could also attempt to replicate the pattern of results observed in the present study in new or existing cross-sectional data among unhappily married dyads. Such an approach could extend the present research by identifying distinct types of unhappy marriages, rather than unhappily married individuals. Further, due to the nature of our dataset, we were unable to examine couple-level processes or outcomes. Researchers could also investigate “types” of unhappy marriages using qualitative and mixed-methods approaches. Such research would add nuance and depth to the present findings and would help guide future research.

Second, these data are cross-sectional and thus we are unable to determine the causal direction and consequential outcomes of the profiles identified in this research. Our analyses cannot determine the extent to which low marital satisfaction is influenced by the indicator variables (i.e., satisfaction with life, family, and friendships), individual differences (e.g., self-esteem and neuroticism), or the interaction between them. Future studies (or secondary data analysis of existing data) could replicate our approach longitudinally to see if profile membership differentially predicts consequential outcomes such as divorce or symptoms of mental health disorders. We suspect, for example, that participants with low marital satisfaction but average satisfaction with their lives, family, and friendships, would be less likely to get divorced than those who are globally dissatisfied. Given the cross-sectional nature of these data, it could also be the case that participants’ responses were impacted by transient feelings or circumstances not directly related to their marriage such as their mood, a stressful life event, or physical health. Such influences could be mitigated in future longitudinal research.

Finally, future research could also investigate the dyadic dynamics of these profiles, such as the extent to which unhappily married couples might be split between the globally dissatisfied and partially satisfied profiles. Previous longitudinal work has investigated the implications of spouses who are “mismatched” in terms of marital satisfaction trajectories (Lavner and Bradbury, 2010; Williamson and Lavner, 2020), but many open questions remain. Future research could extend this work by analyzing subgroups of unhappily married spouses who are categorized into discrepant profiles. In sum, rather than focusing on specific cut points of marital satisfaction when studying discordant marriages, future research could focus on identifying other meaningful indicators of subgroups among unhappily married individuals. Such an approach could inform targeted clinical interventions designed for those who need them most.

## 6 Conclusion

The present study supports Tolstoy (2004) well-known assertion that unhappy families—in this case, spouses—are unhappy in their own way, finding preliminary evidence for two unique subgroups of unhappily married individuals who significantly differed in terms of their satisfaction across life domains. A sensitivity analysis revealed that these subgroups were present at different levels of marital satisfaction within our sample (i.e., the lowest 20%, 22.5%, 25%, 31%, and 34%) and that the patterns of indicator variables between the two groups were remarkably similar at varying levels of marital satisfaction. We hope the patterns identified in this research will help inform future empirical and applied work.

## Data Availability

The dataset presented in this article is not readily available because we do not have ethics approval to upload it online. Requests to access data should be directed to LW at lisawalsh08@gmail.com.

## Footnotes

1
https://www.dynata.com
