# Supplementary material for: Contextualizing marital dissatisfaction: examining profiles of discordant spouses across life domains
Source: Front Psychol. 2025 Mar 19;16:1458129. doi: 10.3389/fpsyg.2025.1458129 (PMC11961942; doi:10.3389/fpsyg.2025.1458129)
Supplement: Supplementary file 1 [file Supplementary_file_1.docx]

**Contextualizing Marital Dissatisfaction: Examining Profiles of Discordant Spouses Across Life Domains**

Annie Regan, Lisa C. Walsh, Calen Horton, Anthony Rodriguez, and Victor A. Kaufman

**SUPPLEMENTARY MATERIALS**

**Contents**

[Tables 2](#_Toc186809039)

[Table S1 2](#_Toc186809040)

[Table S2 3](#_Toc186809041)

[Table S3 4](#_Toc186809042)

[Table S4 5](#_Toc186809043)

[Table S5 6](#_Toc186809044)

[Table S6 7](#_Toc186809045)

[Latent Profile Analysis: Indicator Patterns By Subset 8](#_Toc186809046)

[BCH Procedure: Patterns by Profile 11](#_Toc186809047)

[BCH Procedure: Patterns by Subset 12](#_Toc186809048)

# **Tables**

| **Table S1** | | | | | | |
| --- | --- | --- | --- | --- | --- | --- |
| *Cronbach's Alpha by Subset* | | | | | | |
| **Variable** | **Married Participants** | **Lowest 20% of Marital Satisfaction** | **Lowest 22.5%**  **of Marital Satisfaction** | **Lowest 25% of Marital Satisfaction** | **Lowest 31% of Marital Satisfaction** | **Lowest 34% of Marital Satisfaction** |
| Outcome |  |  |  |  |  |  |
| Marital Satisfaction | 0.98 | 0.95 | 0.96 | 0.96 | 0.96 | 0.96 |
| Indicators |  |  |  |  |  |  |
| Life Satisfaction (SWLS) | 0.90 | 0.88 | 0.88 | 0.88 | 0.88 | 0.89 |
| Life Satisfaction (PWI) | 0.93 | 0.92 | 0.92 | 0.92 | 0.92 | 0.92 |
| Life satisfaction (composite) | 0.94 | 0.93 | 0.93 | 0.93 | 0.93 | 0.93 |
| Family satisfaction | 0.95 | 0.93 | 0.93 | 0.93 | 0.93 | 0.93 |
| Friendship Satisfaction | 0.96 | 0.96 | 0.96 | 0.96 | 0.96 | 0.96 |
| Covariates |  |  |  |  |  |  |
| Perceived Stress | 0.73 | 0.78 | 0.79 | 0.77 | 0.76 | 0.75 |
| Neuroticism | 0.93 | 0.93 | 0.93 | 0.93 | 0.93 | 0.93 |
| Loneliness | 0.87 | 0.85 | 0.85 | 0.84 | 0.85 | 0.85 |
| Self-esteem | 0.89 | 0.9 | 0.91 | 0.91 | 0.9 | 0.9 |

| **Table S2** |  |  |  |  |  |  |
| --- | --- | --- | --- | --- | --- | --- |
| *Model Fit Indices for Latent Profile Analyses by Subset* | | | | | | |
| Subset | -2LL | AIC | BIC | aBIC | LMRT | VLMRT |
| 20% (N = 212) |  |  |  |  |  |  |
| 1 Profile | 1696.124 | 1708.125 | 1728.264 | 1708.53476 | n/a | n/a |
| **2 Profiles** | **1643.696** | **1663.697** | **1697.263** | **1664.79153** | **0.02** | **0.01** |
| 3 Profiles | 1626.716 | 1654.716 | 1701.708 | 1656.84798 | 0.02 | 0.02 |
| 22.5% (N = 241) |  |  |  |  |  |  |
| 1 Profile | 1927.466 | 1939.466 | 1960.375 | 1939.82497 | n/a | n/a |
| **2 Profiles** | **1862.902** | **1882.902** | **1917.75** | **1883.85852** | **< .001** | **0.00** |
| 3 Profiles | 1847.818 | 1875.818 | 1924.605 | 1877.67641 | 0.07 | 0.06 |
| 25% (N = 258) |  |  |  |  |  |  |
| 1 Profile | 2052.564 | 2064.565 | 2085.882 | 2064.89966 | n/a | n/a |
| **2 Profiles** | **1988.554** | **2008.553** | **2044.083** | **2009.44369** | **0.01** | **0.01** |
| 3 Profiles | 1972.16 | 2000.16 | 2049.902 | 2001.8884 | 0.11 | 0.10 |
| 31% (N = 332) |  |  |  |  |  |  |
| 1 Profile | 2656.178 | 2668.178 | 2691.009 | 2668.43646 | n/a | n/a |
| **2 Profiles** | **2568.606** | **2588.606** | **2626.658** | **2589.29136** | **0.01** | **0.01** |
| 3 Profiles | 2547.194 | 2575.194 | 2628.466 | 2576.51892 | 0.49 | 0.48 |
| 34% (N = 364) |  |  |  |  |  |  |
| 1 Profile | 2944.766 | 2956.767 | 2980.15 | 2957.00229 | n/a | n/a |
| **2 Profiles** | **2839.982** | **2859.981** | **2898.953** | **2860.60423** | **<.001** | **<.001** |
| 3 Profiles | 2804.062 | 2832.062 | 2886.622 | 2833.26544 | 0.02 | 0.02 |
| 4 Profiles | 2780.584 | 2816.583 | 2886.732 | 2818.56561 | 0.09 | 0.08 |

*Note.* -2LL=-2 log-likelihood value; AIC=Akaike Information Criterion; BIC= Bayesian Information Criterion; aBIC=Adjusted Bayesian Information Criterion; VLMRT =Vuong-Lo-Mendell-Rubin Likelihood Ratio Test; LRMT=Lo-Mendell-Rubin Test. **Bold** values represent the chosen model/solution.

## **Table S3**

Average Latent Class Probabilities for Most Likely Latent Class Membership

|  | **Profile 1** | **Profile 2** |
| --- | --- | --- |
| **Lowest 20%** | 0.883 | 0.117 |
|  | 0.107 | 0.893 |
| **Lowest 22.5%** | 0.897 | 0.103 |
|  | 0.097 | 0.903 |
| **Lowest 25%** | 0.88 | 0.12 |
|  | 0.1 | 0.9 |
| **Lowest 31%** | 0.897 | 0.103 |
|  | 0.157 | 0.843 |
| **Lowest 34%** | 0.847 | 0.153 |
|  | 0.103 | 0.897 |

*Note.* Profile 1 = Globally dissatisfied (very low friend, family, and life satisfaction). Profile 2 = Partially satisfied (average friendship satisfaction, slightly low life satisfaction, and low family satisfaction).

## **Table S4**

*Descriptive Statistics*

|  | All Married Participants  (n = 1,070) | | | Lowest 20%  (n = 212) | | | Lowest 22.5%  (n = 241) | | | Lowest 25%  (n = 258) | | | Lowest 31%  (n = 332) | | | Lowest 34%  (n = 364) | | |
| --- | --- | --- | --- | --- | --- | --- | --- | --- | --- | --- | --- | --- | --- | --- | --- | --- | --- | --- |
|  | M (SD) | Min | Max | M (SD) | Min | Max | M (SD) | Min | Max | M (SD) | Min | Max | M (SD) | Min | Max | M (SD) | Min | Max |
| Marital Satisfaction | 62.48(17.45) | 0 | 81 | 33.47(13.11) | 0 | 49 | 35.58(13.56) | 0 | 52 | 36.69(13.77) | 0 | 53 | 40.91(14.49) | 0 | 58 | 42.55(14.8) | 0 | 61 |
| Friendship Satisfaction | 2.99(1.2) | 0 | 5 | 2.39(1.15) | 0 | 5 | 2.39(1.16) | 0 | 5 | 2.4(1.15) | 0 | 5 | 2.48(1.15) | 0 | 5 | 2.53(1.17) | 0 | 5 |
| Family Satisfaction | 3.59(0.86) | 1 | 5 | 2.83(0.78) | 1 | 4.9 | 2.87(0.77) | 1 | 4.9 | 2.9(0.76) | 1 | 4.9 | 3(0.77) | 1 | 5 | 3.04(0.78) | 1 | 5 |
| Life Satisfaction (PWI) | 7.38(1.96) | 0 | 10 | 5.66(2.2) | 0 | 10 | 5.74(2.19) | 0 | 10 | 5.75(2.17) | 0 | 10 | 6.03(2.13) | 0 | 10 | 6.16(2.12) | 0 | 10 |
| Life Satisfaction (SWLS) | 25.86(6.42) | 5 | 35 | 20.15(6.95) | 5 | 35 | 20.51(6.89) | 5 | 35 | 20.58(6.82) | 5 | 35 | 21.28(6.73) | 5 | 35 | 21.68(6.76) | 5 | 35 |
| Loneliness | 17.33(4.59) | 8 | 32 | 20.24(4.35) | 8 | 32 | 20.07(4.33) | 8 | 32 | 20.01(4.33) | 8 | 32 | 19.71(4.43) | 8 | 32 | 19.56(4.48) | 8 | 32 |
| Self-Esteem | 32.75(5.87) | 10 | 40 | 29.04(6.33) | 10 | 40 | 29.17(6.32) | 10 | 40 | 29.26(6.28) | 10 | 40 | 29.77(6.08) | 10 | 40 | 29.81(6.01) | 10 | 40 |
| Neuroticism | 6.92(6.39) | 0 | 23 | 11.04(7.15) | 0 | 23 | 10.66(7.04) | 0 | 23 | 10.56(7) | 0 | 23 | 9.85(6.98) | 0 | 23 | 9.65(6.97) | 0 | 23 |
| Health | 2.5(0.97) | 1 | 5 | 2.98(0.97) | 1 | 5 | 2.93(0.97) | 1 | 5 | 2.93(0.96) | 1 | 5 | 2.9(0.95) | 1 | 5 | 2.84(0.96) | 1 | 5 |
| Perceived Stress | 4.98(3.18) | 0 | 16 | 7.12(3.17) | 0 | 16 | 7.01(3.13) | 0 | 16 | 7.02(3.07) | 0 | 16 | 6.61(3.1) | 0 | 16 | 6.58(3.07) | 0 | 16 |

*Note.* Descriptive statistics for unstandardized variables by subset.

**Table S5.** *Relationship Between Profile Membership and Categorical Variables*

|  | **20% Subset** | | **22.5% Subset** | | **25% Subset** | | **31% Subset** | | **34% Subset** | |
| --- | --- | --- | --- | --- | --- | --- | --- | --- | --- | --- |
| **Age** | Profile 1 | Profile 2 | Profile 1 | Profile 2 | Profile 1 | Profile 2 | Profile 1 | Profile 2 | Profile 1 | Profile 2 |
| 18 to 34 | 0.13 | 0.11 | 0.14 | 0.11 | 0.13 | 0.10 | 0.11 | 0.14 | 0.15 | 0.11 |
| 35 to 44 | 0.28 | 0.23 | 0.28 | 0.20 | 0.28 | 0.22 | 0.24 | 0.26 | 0.26 | 0.24 |
| 34 to 54 | 0.30 | 0.24 | 0.28 | 0.25 | 0.30 | 0.24 | 0.23 | 0.31 | 0.30 | 0.24 |
| 55 to 64 | 0.18 | 0.28 | 0.18 | 0.30 | 0.18 | 0.29 | 0.30 | 0.17 | 0.17 | 0.28 |
| 65 and over | 0.12 | 0.15 | 0.12 | 0.15 | 0.12 | 0.14 | 0.13 | 0.12 | 0.12 | 0.13 |
|  |  |  |  |  |  |  |  |  |  |  |
| **Gender** |  |  |  |  |  |  |  |  |  |  |
| Male | 0.38 | 0.45 | 0.37 | 0.47 | 0.39 | 0.45 | 0.42 | 0.48 | 0.43 | 0.49 |
| Female | 0.62 | 0.55 | 0.63 | 0.54 | 0.61 | 0.55 | 0.58 | 0.52 | 0.57 | 0.52 |
|  |  |  |  |  |  |  |  |  |  |  |
| **Education** |  |  |  |  |  |  |  |  |  |  |
| High school or less | 0.19 | 0.10 | 0.19 | 0.10 | 0.17 | 0.10 | 0.18 | 0.09 | 0.19 | 0.10 |
| Some college | 0.22 | 0.18 | 0.22 | 0.19 | 0.23 | 0.19 | 0.21 | 0.20 | 0.21 | 0.19 |
| College graduate | 0.42 | 0.43 | 0.43 | 0.43 | 0.44 | 0.41 | 0.46 | 0.37 | 0.46 | 0.38 |
| Post graduate | 0.17 | 0.30 | 0.16 | 0.29 | 0.16 | 0.30 | 0.15 | 0.35 | 0.15 | 0.33 |
|  |  |  |  |  |  |  |  |  |  |  |
| **Income** |  |  |  |  |  |  |  |  |  |  |
| Less than $30,000 | 0.11 | 0.04 | 0.11 | 0.05 | 0.11 | 0.05 | 0.12 | 0.05 | 0.12 | 0.06 |
| $30,000-$49,999 | 0.19 | 0.11 | 0.19 | 0.11 | 0.19 | 0.10 | 0.20 | 0.09 | 0.20 | 0.08 |
| $50,000-$74,999 | 0.11 | 0.14 | 0.12 | 0.15 | 0.13 | 0.15 | 0.13 | 0.14 | 0.14 | 0.13 |
| $75,000-$99,999 | 0.18 | 0.13 | 0.19 | 0.12 | 0.19 | 0.13 | 0.19 | 0.15 | 0.19 | 0.17 |
| $100,000-$149,999 | 0.19 | 0.21 | 0.19 | 0.21 | 0.18 | 0.22 | 0.18 | 0.22 | 0.18 | 0.23 |
| $150,000 or greater | 0.22 | 0.37 | 0.21 | 0.36 | 0.21 | 0.34 | 0.18 | 0.35 | 0.18 | 0.34 |
|  |  |  |  |  |  |  |  |  |  |  |
| **Best Friend** |  |  |  |  |  |  |  |  |  |  |
| Yes | 0.42 | 0.51 | 0.41 | 0.52 | 0.39 | 0.53 | 0.37 | 0.51 | 0.37 | 0.52 |
| No, multiple close friends | 0.21 | 0.34 | 0.23 | 0.34 | 0.23 | 0.33 | 0.22 | 0.35 | 0.23 | 0.35 |
| No best friend | 0.37 | 0.15 | 0.36 | 0.14 | 0.38 | 0.14 | 0.40 | 0.14 | 0.40 | 0.13 |

*Note.* Profile 1 = Globally dissatisfied (very low friend, family, and life satisfaction). Profile 2 = Partially satisfied (average friendship satisfaction, slightly low life satisfaction, and low family satisfaction).

**Table S6.** *Correlations*

| ***Married Participants (N = 1,070)*** | **1** | **2** | **3** | **4** | **5** | **6** | **7** | **8** | **9** |
| --- | --- | --- | --- | --- | --- | --- | --- | --- | --- |
| 1. Marital Satisfaction | – |  |  |  |  |  |  |  |  |
| 2. Life Satisfaction | .58*** | – |  |  |  |  |  |  |  |
| 3. Family Satisfaction | .57*** | .56*** | – |  |  |  |  |  |  |
| 4. Friendship Satisfaction | .33*** | .44*** | .38*** | – |  |  |  |  |  |
| 5. Neuroticism | -.38*** | -.44*** | -.40*** | -.17*** | – |  |  |  |  |
| 6. Loneliness | -.39*** | -.36*** | -.34*** | -.20*** | .55*** | – |  |  |  |
| 7. Health | .33*** | .51*** | .32*** | .20*** | -.33*** | -.17*** | – |  |  |
| 8. Self-Esteem | .39*** | .53*** | .39*** | .18*** | -.62*** | -.52*** | .35*** | – |  |
| 9. Stress | -.42*** | -.56*** | -.42*** | -.16*** | .59*** | .46*** | -.34*** | -.66*** | – |
| ***Lowest 20% (n = 212)*** | **1** | **2** | **3** | **4** | **5** | **6** | **7** | **8** | **9** |
| 1. Marital Satisfaction | – |  |  |  |  |  |  |  |  |
| 2. Life Satisfaction | .35*** | – |  |  |  |  |  |  |  |
| 3. Family Satisfaction | .41*** | .33*** | – |  |  |  |  |  |  |
| 4. Friendship Satisfaction | .25*** | .38*** | .27*** | – |  |  |  |  |  |
| 5. Neuroticism | -.23*** | -.51*** | -.37*** | -.25*** | – |  |  |  |  |
| 6. Loneliness | -.20** | -.34*** | -.40*** | -.25*** | .52*** | – |  |  |  |
| 7. Health | .13 | .40*** | .19** | .07 | -.36*** | -.10 | – |  |  |
| 8. Self-Esteem | .13* | .60*** | .31*** | .21** | -.63*** | -.40*** | .38*** | – |  |
| 9. Stress | -.18** | -.59*** | -.33*** | -.16* | .60*** | .29*** | -.33*** | -.68*** | – |
| ***Lowest 22.5% (n = 241)*** | **1** | **2** | **3** | **4** | **5** | **6** | **7** | **8** | **9** |
| 1. Marital Satisfaction | – |  |  |  |  |  |  |  |  |
| 2. Life Satisfaction | .35*** | – |  |  |  |  |  |  |  |
| 3. Family Satisfaction | .41*** | .32*** | – |  |  |  |  |  |  |
| 4. Friendship Satisfaction | .21*** | .37*** | .29*** | – |  |  |  |  |  |
| 5. Neuroticism | -.26*** | -.50*** | -.38*** | -.26*** | – |  |  |  |  |
| 6. Loneliness | -.21*** | -.35*** | -.37*** | -.27*** | .50*** | – |  |  |  |
| 7. Health | .17** | .40*** | .19** | .04 | -.34*** | -.09 | – |  |  |
| 8. Self-Esteem | .14* | .58*** | .28*** | .21** | -.62*** | -.41*** | .36*** | – |  |
| 9. Stress | -.19** | -.59*** | -.33*** | -.17** | .57*** | .28*** | -.33*** | -.65*** | – |
| ***Lowest 25% (n = 258)*** | **1** | **2** | **3** | **4** | **5** | **6** | **7** | **8** | **9** |
| 1. Marital Satisfaction | – |  |  |  |  |  |  |  |  |
| 2. Life Satisfaction | .33*** | – |  |  |  |  |  |  |  |
| 3. Family Satisfaction | .43*** | .31*** | – |  |  |  |  |  |  |
| 4. Friendship Satisfaction | .21*** | .36*** | .29*** | – |  |  |  |  |  |
| 5. Neuroticism | -.26*** | -.50*** | -.37*** | -.24*** | – |  |  |  |  |
| 6. Loneliness | -.21*** | -.34*** | -.36*** | -.26*** | .50*** | – |  |  |  |
| 7. Health | .16* | .41*** | .18** | .04 | -.35*** | -.10 | – |  |  |
| 8. Self-Esteem | .14* | .58*** | .28*** | .19** | -.63*** | -.41*** | .37*** | – |  |
| 9. Stress | -.18** | -.59*** | -.31*** | -.15* | .57*** | .28*** | -.33*** | -.65*** | – |
| ***Lowest 31% (n = 212)*** | **1** | **2** | **3** | **4** | **5** | **6** | **7** | **8** | **9** |
| 1. Marital Satisfaction | – |  |  |  |  |  |  |  |  |
| 2. Life Satisfaction | .37*** | – |  |  |  |  |  |  |  |
| 3. Family Satisfaction | .44*** | .35*** | – |  |  |  |  |  |  |
| 4. Friendship Satisfaction | .22*** | .35*** | .31*** | – |  |  |  |  |  |
| 5. Neuroticism | -.29*** | -.46*** | -.33*** | -.19*** | – |  |  |  |  |
| 6. Loneliness | -.22*** | -.32*** | -.33*** | -.24*** | .49*** | – |  |  |  |
| 7. Health | .15** | .39*** | .22*** | .06 | -.32*** | -.08 | – |  |  |
| 8. Self-Esteem | .19*** | .57*** | .28*** | .18*** | -.61*** | -.41*** | .33*** | – |  |
| 9. Stress | -.26*** | -.60*** | -.32*** | -.15** | .57*** | .32*** | -.28*** | -.65*** | – |
| ***Lowest 34% (n = 364)*** | **1** | **2** | **3** | **4** | **5** | **6** | **7** | **8** | **9** |
| 1. Marital Satisfaction | – |  |  |  |  |  |  |  |  |
| 2. Life Satisfaction | .40*** | – |  |  |  |  |  |  |  |
| 3. Family Satisfaction | .44*** | .39*** | – |  |  |  |  |  |  |
| 4. Friendship Satisfaction | .25*** | .38*** | .32*** | – |  |  |  |  |  |
| 5. Neuroticism | -.30*** | -.44*** | -.31*** | -.16** | – |  |  |  |  |
| 6. Loneliness | -.23*** | -.31*** | -.31*** | -.22*** | .49*** | – |  |  |  |
| 7. Health | .20*** | .40*** | .25*** | .08 | -.32*** | -.09 | – |  |  |
| 8. Self-Esteem | .18*** | .52*** | .27*** | .15** | -.61*** | -.38*** | .32*** | – |  |
| 9. Stress | -.25*** | -.57*** | -.31*** | -.12* | .56*** | .31*** | -.28*** | -.65*** | – |

*Note.* *p < .05; **p < .01; ***p < .001.

# **Latent Profile Analysis: Indicator Patterns By Subset**

Profile 1: Globally dissatisfied (very low friend, family, and life satisfaction)

Profile 2: Partially satisfied (average friendship satisfaction, slightly low life satisfaction, and low family satisfaction)


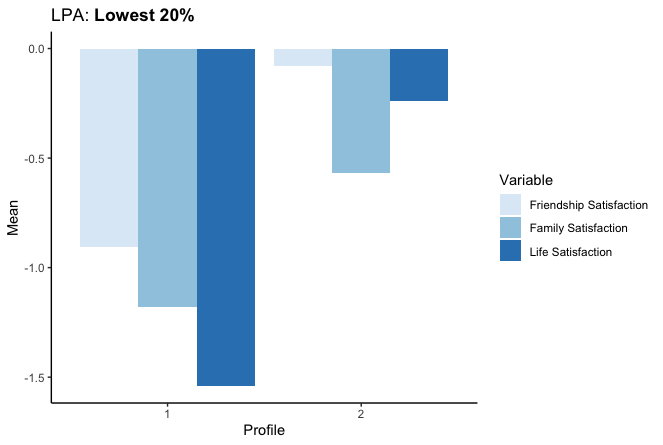


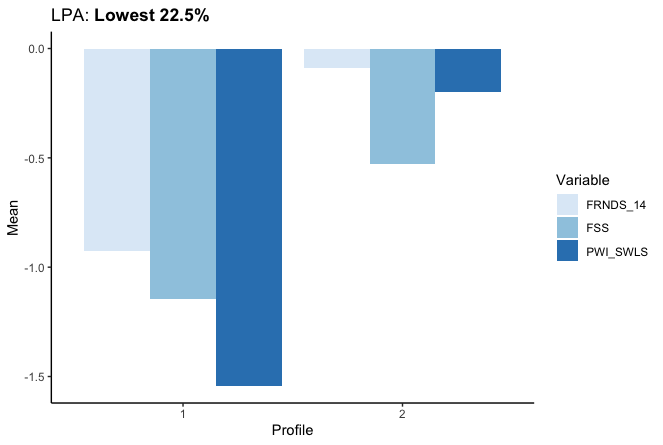

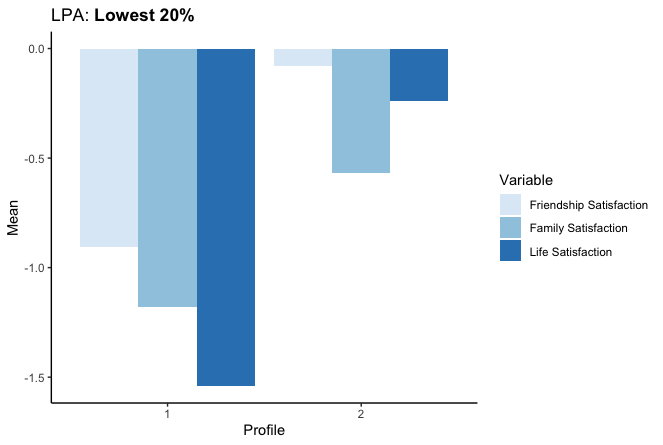


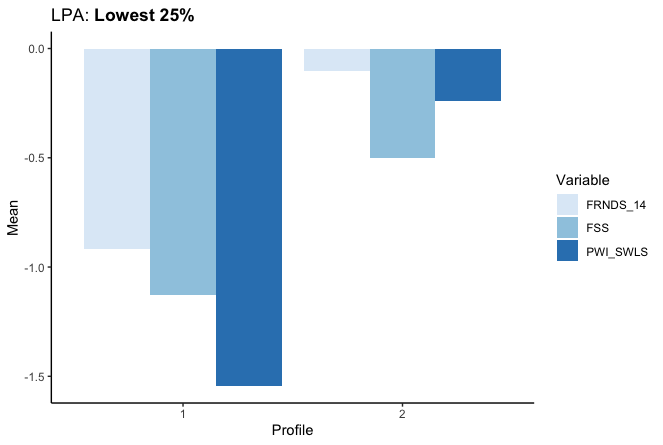

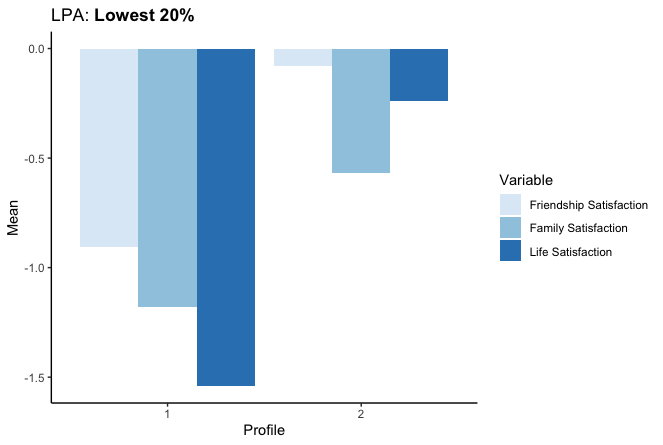


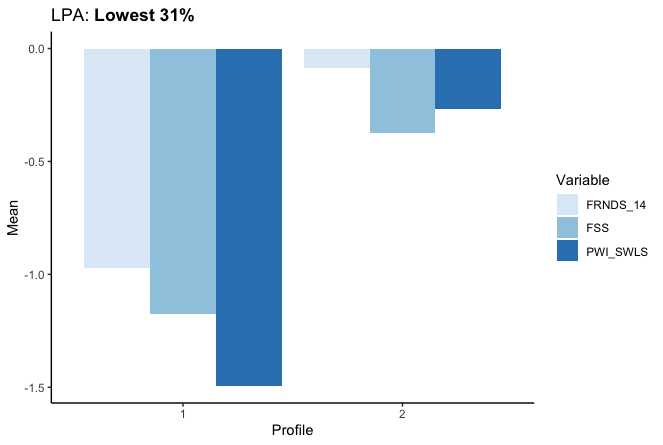

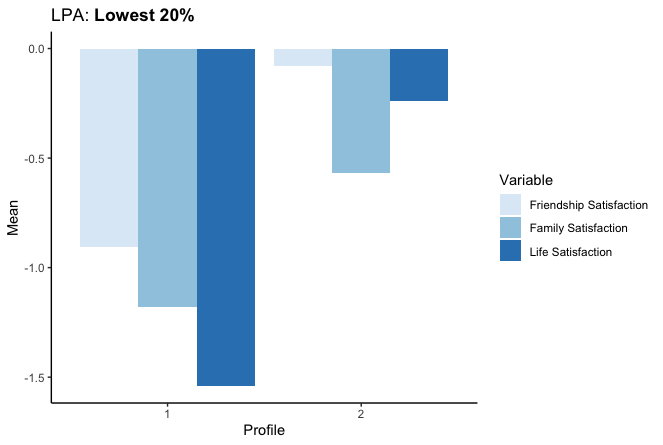


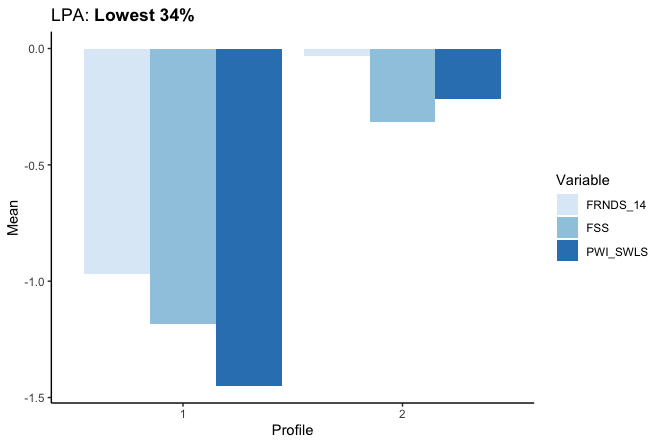

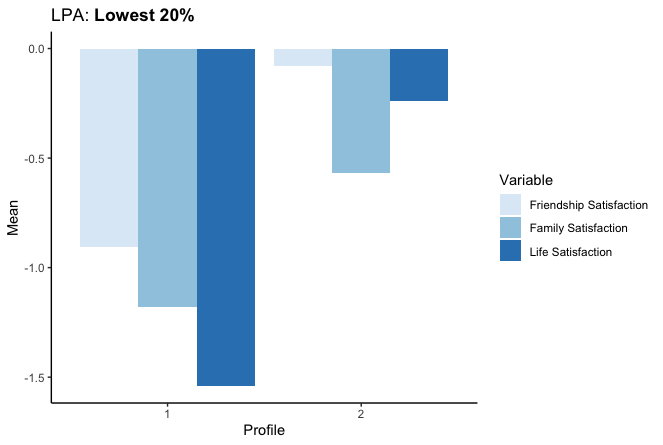


# **BCH Procedure: Patterns by Profile**


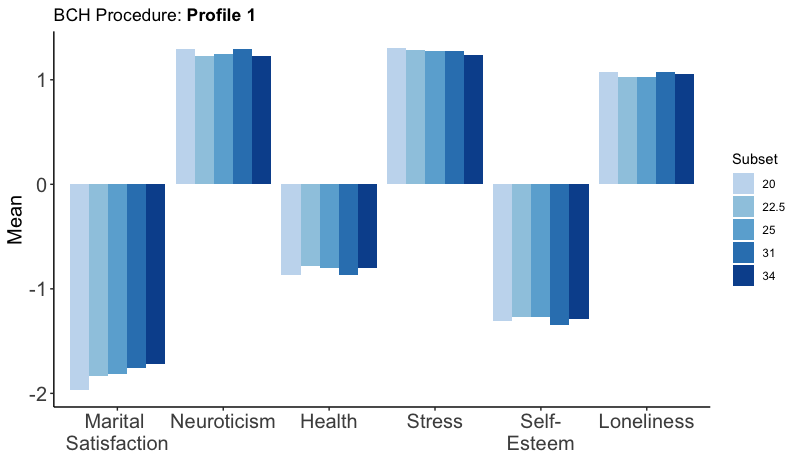


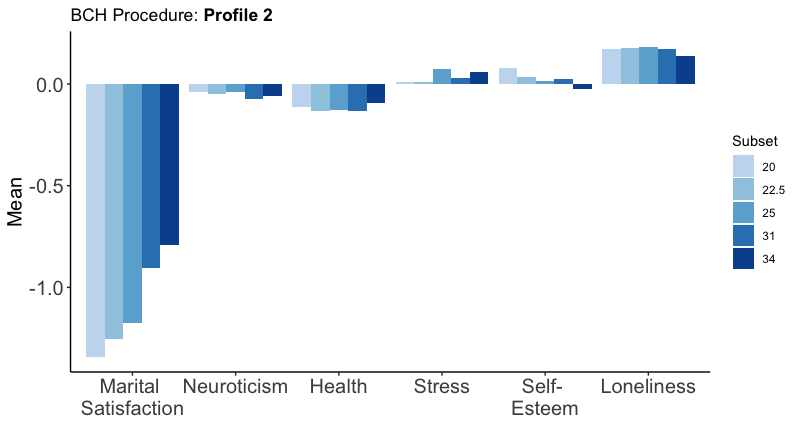


# **BCH Procedure: Patterns by Subset**

Profile 1: Globally dissatisfied (very low friend, family, and life satisfaction)

Profile 2: Partially satisfied (average friendship satisfaction, slightly low life satisfaction, and low family satisfaction)

**
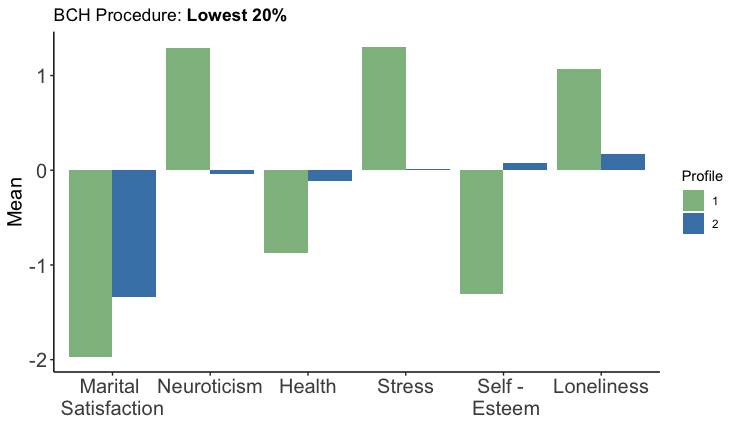
**

**
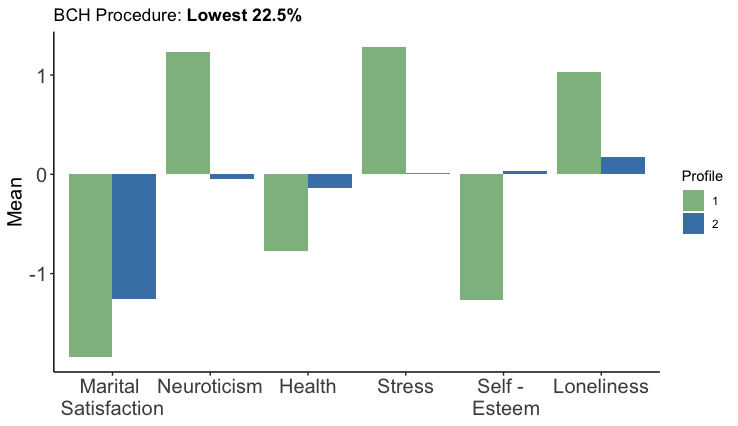
**

**
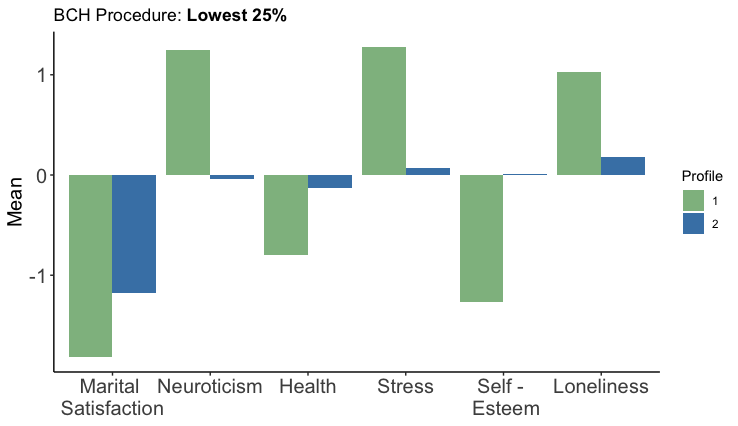
**

**
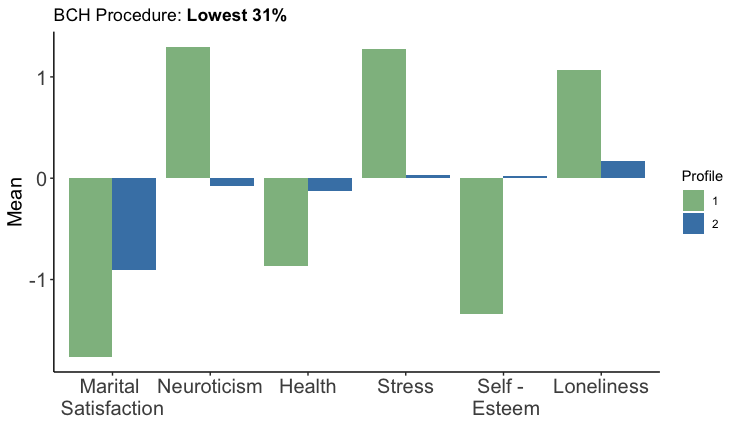
**

**
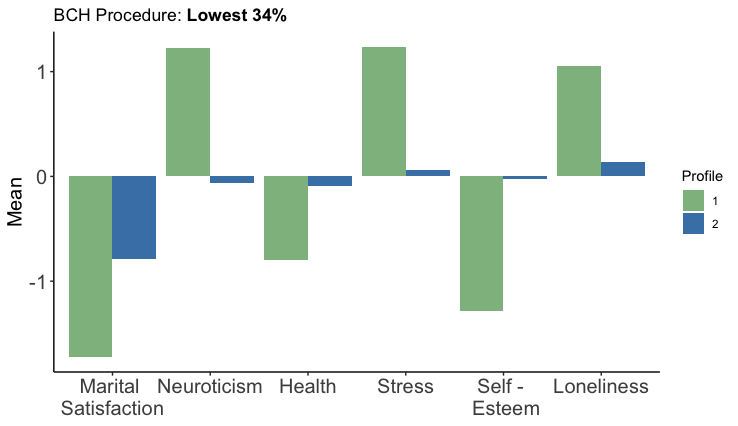
**
